# Aging of adult lifetime survivors with perinatal HIV

**DOI:** 10.1097/COH.0000000000000938

**Published:** 2025-05-21

**Authors:** Indira Mallik, Merle Henderson, Sarah Fidler, Caroline Foster

**Affiliations:** a Imperial College Healthcare Trust; b Department of Infectious Disease, Faculty of Medicine, Imperial College London; c Imperial College NIHR BRC, London UK

**Keywords:** comorbidities, lifelong HIV, mental health, perinatally acquired HIV, sexual health and pregnancy, young adults

## Abstract

**Purpose of review:**

We describe the emerging clinical outcomes for adults born with perinatally acquired HIV (PHIV), who have been living with HIV throughout their life. Whilst many comorbidities appear similar to adults with horizontally acquired HIV, they manifest at a younger chronological age. The additional impact of HIV throughout postnatal, childhood and adolescent growth and development requires further consideration.

**Recent findings:**

There is growing evidence of an increased incidence of metabolic, cardiovascular, respiratory, bone and renal impairment as well as structural brain changes associated with impaired cognitive function, and mental health disorders; early case series data suggests a six-fold increased prevalence of psychosis for those with lifelong HIV compared with age-matched peers. Older age, prior CDC-C diagnoses and lower nadir CD4 count confer the greatest risk of PHIV complications in adulthood, but biological factors are compounded by socioeconomic deprivation, bereavement, HIV-associated stigma, discrimination and immigration. The aetiology of these increased comorbidities is yet to be fully elucidated but includes lifelong systemic inflammation and immune dysfunction despite suppressive antiretroviral therapy (ART).

**Summary:**

Adults living with lifelong HIV experience increased risk of comorbidities at a younger chronological age despite viral suppression on ART. Exploring the aetiology and characterizing the clinical manifestations of lifelong HIV can best inform screening tools and interventions that can enhance quality of life and longevity.

## INTRODUCTION

An estimated 11 million children have been born with perinatally acquired HIV (PHIV) since 1990, over 80% in the sub-Saharan region of Africa [1]. Despite advances in prevention of vertical transmission since the peak in 2000, annually 120 000 (83 000–170 000) children continue to acquire HIV [1]. The global roll out of antiretroviral therapy (ART) has dramatically improved survival for all people living with HIV and in settings where children with PHIV received suppressive ART from the late 1990s including the United States and Europe, the oldest survivors are entering their fifth decade of life [2].

Adults living with lifelong perinatal HIV (APHIV), like all adults who grow up with chronic illness in childhood, may have to contend with frequent hospitalizations in childhood and difficulties of taking regular medication from a young age, and as a result may experience a degree from isolation from peers who do not have a chronic illness. In addition, those who grow up with lifelong HIV frequently encounter HIV-related stigma in the home, community and in healthcare settings, as well as parental bereavement, deprivation, and in some cases, insecure immigration status. Dealing with such a wide array of medical and psychosocial issues makes transition from paediatric to adult services in late adolescence a particularly vulnerable time [2], but ageing with lifelong HIV is complex beyond this life stage. 

**Box 1:**
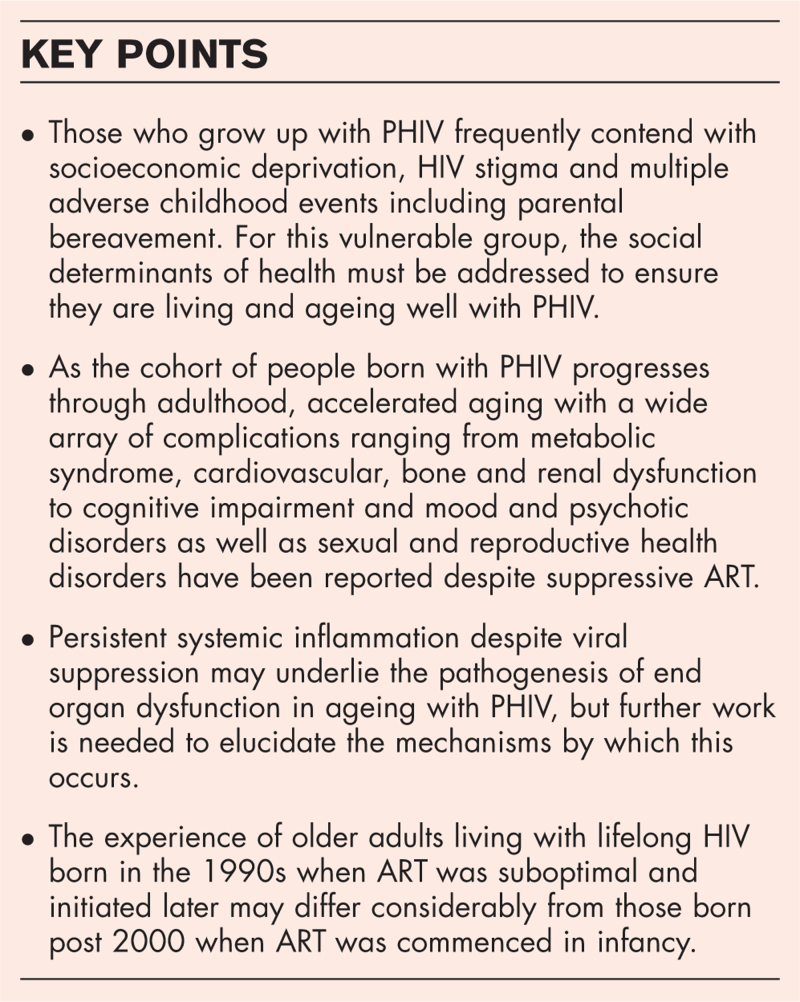
no caption available

Emerging data suggests APHIV are at increased risk of non-AIDS complications compared to age-matched populations without HIV, despite viral suppression on ART, thought to be a consequence of HIV-associated inflammation [3] and immune activation [4]. Complications typically associated with ageing are reported in young adults with PHIV and include [5] neurocognitive decline [6^▪▪^], increased rates of cardiovascular and metabolic [7], respiratory [8], renal [9] and liver [10] diseases (Table 1). The underlying mechanisms remain complex and may differ by age cohort. The current cohort of PHIV adults, born in the 1990s, typically initiated treatment in mid childhood with suboptimal and more toxic ART agents [2,5]. However, this cohort survived early childhood without ART and may be enriched for favourable host genetics and immune responses [4]. In contrast, the emerging cohort of those born after 2000 increasingly started ART in early childhood; their outcomes may differ. In this review, we update the field on the current reported clinical data amongst adults living with lifelong HIV.

**Table 1:** Common complications in adults with perinatally acquired HIV and potential interventions

Complication	Risk factors	Suggested intervention	Evidence
Metabolic syndrome including hyperlipidaemia, diabetes mellitus and steatotic liver disease	Obesity Poor diet Lack of exercise Prolonged exposure to protease inhibitors Past CDC-C diagnosis	Healthy lifestyle interventions including referral to dieticians and weight reduction strategies, including considering GLP-1 receptor agonists Optimize ART regimens Limit protease inhibitor exposure wherever possible	Haw *et al.*[17^▪▪^] Dirajlal-Fargo *et al.*[20] Rose *et al.*[24] Lahiri *et al.*[26]
Atherosclerotic cardiovascular disease	Obesity Hypertension Hyperglycaemia Dyslipidaemia Smoking history Sustained or transient viraemia Past CDC-C diagnoses Low nadir CD4 Years of viraemia ART regimen (boosted PI and integrase inhibitors)	Early assessment and risk stratification for APHIV younger than 40 years using validated tools such as PDAY Lifestyle modifications Optimize ART regimen Statins Fibrates Consider aspirin prophylaxis	Greybe *et al.*[34^▪^] Mahtab *et al.*[33]
Impairment in executive functioning, processing speed and working memory	Late ART initiation Low nadir CD4 count Past CDC- C diagnoses, particularly HIV encephalopathy	Maintain viral suppression	Dahmani *et al.*[6^▪▪^]
Mood and psychotic disorders	Genetic Past CDC- diagnoses Low nadir CD4 count Presence of learning disabilities Migrant status Adverse childhood events including parental bereavement Substance misuse (particularly cannabis use) ART: efavirenz, INSTI	ART optimization, limit efavirenz use Encourage abstinence from cannabis Integration of psychology and peer support services into routine HIV care Long-term injectable ART may be useful in maintaining adherence to ART	Mallik *et al.*[43] Mudra Rakshasa-Loots *et al.*[56^▪▪^]
Renal dysfunction	Black ethnicity Persistent viraemia Hypertension Hyperglycaemia	Optimizing ART regimen (using TAF where indicated) Managing hypertension, and lowering HbA1c	Haw *et al.*[17^▪▪^] Nasuuna *et al.*[70^▪^] Diana and Naicker [72]
Low bone mineral density	Late ART initiation Duration of ART use Older age Female sex Physical inactivity Malnutrition/low BMI Vitamin D deficiency	Consider switch to TAF regimens Monitor Vitamin D levels and supplement if needed	Sudjaritruk *et al*. [82] Henderson *et al.*[80]
Increased risk of anogenital HPV infection High rates of sexually transmitted illnesses		Encourage routine STI screening as part of HIV care Encourage vaccination; HPV, HBV, HAV, M Pox, meningococcus Encourage engagement with cervical and/or anal screening programmes	Murahwa *et al.*[96] Jongen *et al.*[97^▪^] Nott *et al.*[87]

APHIV, adults with perinatally acquired HIV; ART, antiretroviral therapy; GLP-1, glucagon-like peptide-1; HAV, hepatitis A virus; HBV, hepatitis B virus; HPV, human papilloma virus; INSTI, integrase strand transfer inhibitor; PHIV, perinatally acquired HIV; PI, protease inhibitor; STI, sexually transmitted infection; TAF, tenofovir alafenamide.

## IMPACT OF LIFELONG ART AND FUTURE CONSIDERATIONS

Rates of viral suppression are lower in children and adolescents who have grown up with lifelong HIV compared to adults with horizontally acquired HIV, resulting in higher rates of acquired drug resistance [11]. In many settings, nonnucleoside reverse transcriptase inhibitors (NNRTI) were first-line ‘3rd’ agents for paediatric ART prior to the introduction of second-generation integrase strand transfer inhibitors (INSTI). In a UK study of cumulative drug resistance in 280 APHIV; median age 26 years and duration on ART 17 years; 78% had prior NNRTI exposure and 37% NNRTI resistance [12^▪^]. Factors associated with cumulative drug resistance mutations were duration on ART and prior NRTI mono or dual therapy [12^▪^]. These findings are consistent with data from other PHIV cohorts globally [13,14]. As a result, currently approved long-acting injectable ART (cabotegravir/rilpivirine) is unsuitable for a significant proportion of APHIV. Alternative injectable treatment options should be prioritized in this cohort, especially those struggling to take oral medications. A recent case-series of 34 viraemic adults, reported 59% with NNRTI resistance, but high-rates of virological suppression with injectable cabotegavir/lenacapavir [15].

## METABOLIC HEALTH

Early data suggests APHIV are at increased risk of adverse metabolic health compared to aged matched peers living without HIV [5,16]. Risk factors include lifelong HIV exposure, chronic immune activation, ART-associated side effects alongside traditional risk factors including diet, raised BMI, lack of exercise and with ethnicity. Increased rates of the metabolic syndrome, type 2 diabetes mellitus and cardiovascular events are reported amongst APHIV compared to aged and ethnically matched HIV-negative controls (HIV−) [5,17^▪▪^]. Cohorts in the United States report incidence rates of type 2 diabetes mellitus of 19%, hypercholestrolaemia 40% and hypertriglyceridaemia 50% by the age of 30 years [17^▪▪^]. Rates of impaired glucose metabolism increased with both age and BMI in adolescents with PHIV in Ghana [18]. This was not reproduced in a Ugandan PHIV cohort, although insulin sensitivity was reduced compared to HIV populations [19]. Whilst obesity is a major driver of insulin resistance and dyslipidaemia, the role of chronic immune activation in glucose homeostasis is emerging, with markers of increased gut permeability in adolescents with PHIV associated with reduced insulin sensitivity despite viral suppression [20,21]. Body fat distribution drives the metabolic syndrome with higher rates of central adiposity in APHIV (median age 26 years) compared to controls despite similar BMI, associated with prolonged exposure to boosted protease inhibitors [22].

Obesity driving metabolic dysfunction-associated steatotic liver disease is the main driver of liver fibrosis in adults with HIV mono-infection [23]. Early data in APHIV reports increased rates of hepatic steatosis compared to HIV− controls, more prevalent with age and only partially explained by metabolic factors [24,25]. Preliminary data suggests improvement in liver disease following switching to dolutegravir from older ART regimens, although the role of INSTIs and weight gain may limit improvement [24,26].

## CARDIOVASCULAR DISEASE

Pre-ART, HIV-related cardiac disease was well recognized in PHIV children [27]. While severe cardiac disease declined with ART use, APHIV remain at higher risk of cardiovascular disease (CVD), compared to age-matched HIV− populations, with demonstrable abnormalities in cardiovascular structure and function [16,28–31]. Subclinical left ventricular (LV) dilation with reduced systolic function and diffuse myocardial fibrosis on cardiac MRI (cMRI) is reported for South African PHIV youth [29]. Individuals with early-ART initiation (<5 years old) had improved LV systolic function and lower LV mass, when compared to delayed ART initiation (>5 years) [29]. However, effects may not be fully ameliorated by ART; a recent cross-sectional analysis of South African youth (287 PHIV, 87 non-PHIV and 90 HIV−; median age 16 (range 11–24) years) demonstrated higher rates of subclinical myocardial fibrosis in those with HIV compared with HIV− controls [30]. Abnormal surrogate markers of atherosclerotic vascular disease are described in APHIV, including increased carotid intima media thickness, arterial stiffness and impaired endothelial function [32]. How these findings correlate with coronary plaque formation and clinical outcomes, remains unexplored.

The mechanisms underlying CVD in PHIV are not fully elucidated and are likely multifactorial; lifelong exposure to HIV and ART, periods of viraemia, chronic immune activation and vascular inflammation. Traditional risk factors further contribute to the pathogenesis potentially exacerbated by ART agents with adverse metabolic profiles [22,33]. The Pathological Determinants of Atherosclerosis in Youth (PDAY) score is validated for HIV− young adults (<40 years) and predicts atherosclerotic CVD risk based on traditional factors including sex, obesity, hypertension, hyperglycaemia, dyslipidaemia and smoking history [24]. In a recent study of 218 South African PHIV (median age 16.8 years), 30% had a raised PDAY score (≥1), associated with both traditional [low high-density lipoprotein (HDL) cholesterol] and HIV-related risk factors (sustained or transient viraemia and ART duration) [33]; findings reflected in other studies [34^▪^]. Acknowledging that APHIV may be at a higher risk of cardiovascular disease from a younger age, CVD risk assessment may help to inform early intervention in high-risk individuals. Importantly, however, such scores may underestimate risk in youth with HIV due to the direct effects of HIV infection and ART exposure.

## NEUROLOGICAL COMPLICATIONS

Despite suppressive ART, some APHIV have impaired cognitive function compared to both HIV-exposed uninfected (HEU) and HIV-unexposed and uninfected (HUU) age-matched groups [6^▪▪^] with higher order functions, particularly executive function, processing speed and working memory negatively affected [35]. These differences are associated with structural brain changes [36], with an ameliorating effect of early ART initiation [37], and the most profound changes associated with lower nadir CD4 counts and a history of CDC-C conditions, particularly infantile HIV encephalopathy [38]. However, present longitudinal studies include APHIV exposed to more neurotoxic ART in childhood. Cognition, particularly higher order function is complex and multifactorial, with socioeconomic deprivation correlating with worse cognitive outcomes in a recent meta-analysis [6^▪▪^]. Furthermore, although most APHIV live in sub-Saharan Africa, studies in neurocognition have predominantly taken place in well resourced settings and transferability of their findings to the next PHIV generation uncertain [6^▪▪^].

## MENTAL HEALTH

APHIV have higher rates of depression and anxiety than the general population globally [39–42], with emerging data that psychotic disorders may be over six times more prevalent than age matched HIV− peers [43].

The cause of this increased prevalence is multifactorial, and APHIV have an intersection of risk factors for poor mental health: lifelong exposure to a neurotropic virus [44], chronic exposure to ART with neuropsychiatric side-effects including INSTIs [45,46] and efavirenz [47], migration [48], racial discrimination [49], HIV stigma [46], parental bereavement and multiple adverse childhood events [50,51]. Several longitudinal studies have found that both HEU and PHIV youth have similarly high rates of psychiatric and substance misuse disorders, higher than reported in HUU populations, suggesting that social factors and the impact of maternal/familial HIV (e.g. parental bereavement/ill health) are important drivers. [52–55].

The precise pathogenesis of psychotic and mood disorders remains unclear; however, immune dysfunction maybe implicated [56^▪▪^]. Robust evidence suggests neuroinflammation is pathogenically linked with depression, anxiety and psychotic disorders [57], including in adolescents [58] in the general population. Systemic inflammation is associated with symptoms of mood disorders [59], and recent neuroimaging studies describe correlations between peripheral inflammation and impaired functional connectivity and brain microstructural changes linked with depression and psychosis [60–62]. Although no studies have explored the relationship between inflammation and mental health outcomes in APHIV, persistent inflammation despite effective ART is well established [4,63].

The impact of poor mental health on APHIV is profound: the transition from paediatric to adult care is an especially vulnerable time [2], and comorbidity with a mood or psychotic disorder is associated with suboptimal engagement with care, poor ART adherence and virological failure in all settings [2,64–66]. A recent longitudinal study of 207 APHIV (mean age 27) reported that 27% had attempted suicide [67].

## RENAL DISORDERS

Chronic kidney disease (CKD), defined as an estimated glomerular filtration rate (eGFR) less than 60 ml/min/1.73 m^2^ for more than 3 months [68], is common in PHIV [69]. In recent US data, 4% of APHIV met international CKD criteria; however, 25% had abnormally low renal function (eGFR <90 ml/min/1.73 m^2^ for >3 months), with incidence increasing with age [17^▪▪^], suggesting that as APHIV age globally, the burden of CKD will increase. Although data from sub–Saharan Africa is limited by heterogeneity in methods of identifying CKD, a pooled prevalence of 12% is reported [70^▪^]. CKD in PHIV has historically been due to immune complex-mediated glomerulonephritides and HIV-associated nephropathy [71]; however, with effective ART, comorbid metabolic illness including hypertension and diabetes [72] and nephrotoxic effects of lifelong ART [73–75] are emerging. Black ethnicity and HIV viraemia are associated with increased risk of CKD [17^▪▪^,^71^]. There is growing concern that climate change: water scarcity, drought, and extreme heat will exacerbate the CKD risk in lower income and middle-income countries [68], where the majority of APHIV live [76].

## BONE HEALTH

Low bone mineral density (BMD), defined as a dual-energy X-ray absorptiometry (DXA) *z*-score of −2 or lower, is a well recognized complication of HIV, with prevalence estimates of between 12.5 and 16.4% in adolescents and young adults with PHIV, with variation according to setting [77,78]. Low BMD during childhood may increase the risk of osteoporosis and fractures in later life.

Low BMD is likely multifactorial, related to both HIV-associated and traditional risk factors including HIV-related immune activation and inflammation, timing and duration of ART, older age, female sex, physical inactivity, low BMI and vitamin D deficiency [5]. Certain ART agents may negatively impact on bone health; tenofovir disoproxil (TDF) use in Asian adolescents with PHIV aged 10–18 years has been associated with dysregulated bone turnover [78]. However, reassuringly, over 4 years of follow-up, there was no significant associations between low BMD and TDF use or duration [79], and in a UK cohort of APHIV, longitudinal BMD accrual was similar to that of age, sex and ethnicity-matched population data [80].

The effects of vitamin D supplementation on BMD are conflicting. A recent Thai study of PHIV adolescents (*n* = 200, median age 16 years) reported an increase in lumbar spine BMD and a decrease in bone metabolism markers over 48 weeks with vitamin D/calcium supplementation (400 IU/1200 mg/day plus or minus ergocalciferol 20 000 IU/week) [81]. Follow-up 3 years after supplementation demonstrated no significant decline in lumbar spine BMD, despite reductions in serum 25-hydroxyvitamin D concentration and an increase in parathyroid hormone [82]. However, these findings are not consistent, and vitamin D supplementation was not found to have a beneficial effect on musculoskeletal health outcomes in a systematic review of 20 studies in children and youth with HIV [83]. A large-scale clinical trial in Africa is ongoing to further determine the effect of vitamin D/calcium supplementation on bone health [84].

## SEXUAL AND REPRODUCTIVE HEALTH

The majority of data suggests APHIV have a similar age of coitarche compared to their HIV-negative peers; however, the burden of HIV stigma prevents a minority from entering any sexual relationships [5,85,86^▪^,^87^]. The potential positive benefits of sharing ones HIV status with friends and partners include social acceptance, emotional support, ART adherence support and stigma reduction, balanced by potential negatives including rejection, recrimination and a wider loss of confidentiality [63,88–91]. For PHIV adults, sharing their HIV status uniquely risks disclosing the status of family members. Despite this, 62% of Ugandan APHIV (18–25 years) shared their status with sexual partners [92^▪^,^93^]. Young people, carers and healthcare professionals all cite the need for evidence-based interventions to support disclosure with early data suggesting improved wellbeing with structured disclosure interventions specific for APHIV that include peer workers [92^▪^,^94^].

High rates of sexually transmitted infections, unplanned pregnancy and termination are reported in PHIV adults, although rates are comparable to HEU peers [5,87]. Those living with HIV are at increased risk of anogenital human papillomavirus (HPV) related disease and whilst vaccination is highly effective, specific questions remain for APHIV, particularly young men [95,96]. Whether reduced HPV vaccine schedules for the general population will provide adequate sustained protection for APHIV is unclear, and the optimal timing of onset of cervical and/or anal screening for this specific population is unknown with no recommendations in global guidelines [96,97^▪^,^98^].

Alarmingly, high lifetime rates of intimate partner violence (IPV), 84% overall, with 65% in the past year, are reported in both APHIV and HEU in the United States [99^▪▪^]. Whilst rates of IPV were lower (37%) in younger South African adolescents (mean 15 years, 76% PHIV), IPV and sexual abuse were strongly associated with poorer ART adherence highlighting the need for IPV screening and interventions integrated within HIV care [100^▪^].

Emerging data for pregnant women with PHIV suggest they are more likely to be younger, have detectable viraemia, immunosuppression and acquired HIV-1 drug resistance mutations when compared to pregnant people with horizontally acquired HIV [101^▪^,^102^–^106^]. Whilst perinatal HIV transmission rates are reassuringly low, increased rates of preterm delivery and low birth weight are reported [102,104]. Longer term outcome data for the ‘3rd generation’ HEU infants born to APHIV are urgently required with early data suggesting potential deficits in speech and language acquisition and poorer growth when compared to HEU born to non-PHIV adults [107,108]. Reports of increased all-cause maternal mortality postpartum amongst APHIV from Spain and the United States [101^▪^,^104^], is alarming and warrants further investigation.

## CONCLUSION

In this article, we have reviewed the recently published literature on non-AIDS clinical conditions affecting the current population of APHIV (Table 1). There appears to be an increased risk of a broad range of end-organ conditions, and an overall increase in risk factors associated with the development in particular of cardiovascular, and metabolic syndromes. US data suggests that by the age of 30, the incidence of diabetes, dyslipidaemia, CKD and hypertension, in adults living with lifelong PHIV is 50% higher than the aged matched general population.

It is important to note that the group of adults who have grown up with perinatal HIV who are now older than 25 years have had a very different clinical experience to those entering adulthood in the last decade following global availability of modern effective ART regimens. The outcomes for this latter population are only just emerging as they enter adulthood but awareness and rapid management of potential clinical complications, modification of ART regimens to limit toxicities and ensuring viral suppression will be key to maximizing healthy ageing in this cohort.

## Acknowledgements

*We wish to thank the patients of the 900 Clinic, Imperial College Healthcare Trust.*

### Financial support and sponsorship

*M.H and S.F have received grants from Imperial NIHR BRC.*

### Conflicts of interest

*There are no conflicts of interest.*

## REFERENCES AND RECOMMENDED READING

Papers of particular interest, published within the annual period of review, have been highlighted as:

▪ of special interest
▪▪ of outstanding interest
